# The Knowledge and Preferences of Parents/Carers of Autistic Children and Young People about Technology Devices

**DOI:** 10.1007/s10803-024-06678-8

**Published:** 2024-12-21

**Authors:** Athanasia Kouroupa, Karen Irvine, Silvana E. Mengoni, Shivani Sharma

**Affiliations:** 1 Division of Psychiatry, University College London, London, UK; 2 School of Life and Medical Sciences, University of Hertfordshire, Hatfield, Hertfordshire UK

**Keywords:** Autism, Technology, Support, Survey, Parents, Carers

## Abstract

This study explored parents’/carers’ knowledge, interest, and preferences towards technology devices as support mediums for autistic children, the reasoning behind any choice and the factors associated with the most preferred technology device. Technology devices were conceptualised as smartphones, iPods, tablets, virtual reality, robots, and ‘other’ for participants to list their own further interpretations of technology devices. Survey data were collected from 267 parents/carers of autistic children aged 2–18 years internationally between May to October 2020. Parents/carers of autistic children and young people were aware of, interested in and mostly preferred the use of tablets because of their convenience and ease of use. They least preferred virtual reality followed by robots due to both being overwhelming, cold, inconvenient to transport and expensive. Robots, in particular, were unknown to respondents. The data suggested that some technology devices as a support medium are not widely known to families of autistic children and young people in support programmes. Technology devices need to be financially approachable and achieve a high standard of design to engage users. Future research should focus on gathering evidence from the autistic community about their preferences and views of technology devices as a medium in autism support programmes.

**Supplementary Information:**

The online version contains supplementary material available at 10.1007/s10803-024-06678-8.

The benefits of early support for autistic children and young people are well-documented, though long-term outcomes vary widely (NICE, 2021). This variability can in part be attributed to differential experience in access to personalised support and services that meaningfully respond to the individual needs and preferences of autistic children, young people and their families. The heterogeneity of autism and the way experiences and needs change with age underscores the necessity of provision that is flexible and personalised over the life course (Clarke, McGauley, & Lord, 2021; Crowe & Salt, 2015). For example, there are autistic individuals with excellent adaptive skills and minimal support needs in the domain of social and communication skills. Equally there are autistic children and young people who experience difficulty in the domain of social and communication skills alongside co-occurring conditions such as epilepsy, attention deficit hyperactivity disorder, and mental health conditions. This adds to the heterogeneity of intervention and therapeutic approaches for personalisation.

There is extensive literature about the range of non-pharmacological interventions available to support autistic children and young people including behavioural, developmental, naturalistic, sensory-based, and animal-mediated approaches with variable outcomes. A recent meta-analysis reported that naturalistic developmental behavioural approaches (e.g., Early Start Denver Model, Joint Attention, Symbolic Play, Engagement, and Regulation or Pivotal Response Treatment) were effective in the development of social and communication skills, language, play and cognitive skills (Sandbank et al., 2020). Naturalistic developmental behavioural approaches focus on developmentally appropriate skill acquisition learning through behavioural approaches (e.g., rewards) in a natural context (e.g., via play). The remaining approaches (e.g., sensory-based or animal mediated) indicated some or little evidence of effectiveness in the examined skills (e.g., adaptive skills, social and communication skills, behaviour that challenges, language, play etc.) (Sandbank et al., 2020). The same meta-analytic review included 10 technology devices/mediums including computers, tablets, DVDs, video games, and robots that targeted social and communication skills and/or emotional development in young autistic children aged up to 8 years of age with no evidence of effectiveness (Sandbank et al., 2020). However, it is important to emphasise that there was limited to absent human interaction in most of the included studies exploring technology devices/mediums.

The wider literature includes evidence showing that when a tablet serving as a speech generating device was integrated in a validated psychosocial support session (e.g., Discrete Trial Training, Joint Attention, Symbolic Play, Engagement, and Regulation and Enhanced Milieu Teaching), autistic children and young people (aged 3–16 years) demonstrated improvements in their social and communication skills (Kasari et al., 2014; Lorah et al., 2022; van der Meer & Rispoli, 2010). This indicates that mobile technology devices may be beneficial mediums if introduced as part of a validated support programme, whereas evidence for standalone approaches is still lacking. Research of this nature is important when thinking about ways in which to augment the impact of autism support programmes, helping healthcare providers continually enhance the quality and impact of care in resource pressured contexts.

Autistic young people aged 14–21 years use technology devices to access online webpages and/or applications, play games, listen to music, chat, access education, keep notes, set online reminders and other activities (Hedges et al., 2018). In parallel, as described earlier, there are minimally verbal autistic children and young people who use tablets as a speech generating device with positive outcomes (Kasari et al., 2014; Lorah et al., 2022; van der Meer & Rispoli, 2010) to supplement or augment natural speech (Beukelman & Mirenda, 1998). Given the general emphasis of digital innovation in international health policy, it is useful to consider how technology can be harnessed for the benefit of autistic individuals (Vazquez-Venegas et al., 2024).

Interventions that use technology devices such as computers, tablets, virtual reality, and robots act as mediators in a session delivered by a trained healthcare professional. These support programmes target the reported interest in technology by many autistic children and young people (Grynszpan et al., 2014). For example, reports indicate that autistic children aged 7 years spent about 50–94 min on tablets daily (Clark et al., 2015). Similar findings about technology use have been reported by parents whose neurotypical children (mean age = 6.27 years) usually spent 60–120 min per day engaging with tablets (Oliemat et al., 2018). There are certain characteristics in technology including accessibility, predictability, controlled use, and engaging nature that may be particularly attractive to autistic children and young people (Diehl et al., 2012; Hedges et al., 2018; Kellems & Morningstar, 2012; Laurie et al., 2019; Pavlopoulou et al., 2022; Sandbank et al., 2020). A recent exploration of technology use in autistic adolescents revealed that access to a smartphone, tablet, computer, and laptop increased their sense of independence, reduced anxiety levels, and promoted opportunities for social engagement (Hedges et al., 2018). There is also evidence that although autistic adolescents usually engage in individual rather than group games, they actively interact with peers via text and/or online messaging in chat rooms during gaming (Kuo et al., 2014). Nonetheless, there are some reported disadvantages in excessive technology use by autistic children and young people, such as hyperactivity, lack of attention, early signs of addiction to technology, and sleep problems (Coutelle et al., 2021; Craig et al., 2021).

Existing evidence around the use of technology devices in autism has predominantly been focused on testing its effectiveness on social and communication skills with promising outcomes albeit requiring more rigorous evidence (Costescu et al., 2014; Grynszpan et al., 2014; Kouroupa et al., 2022; Sandbank et al., 2020; Soares et al., 2021). For instance, smartphones, iPods, computers, and tablets have been introduced in sessions controlled via a healthcare professional have all been used to engage the autistic child’s attention and/or support their mental wellbeing through gaming or video watching (i.e., reward, break, build on strong visual processing skills) though with limited evidence of their effectiveness in the autism literature (Alzrayer et al., 2014; Brunero et al., 2019; Fletcher-Watson et al., 2016; Hillier et al., 2016; Kellems & Morningstar, 2012; Maglione et al., 2012).

During the COVID-19 public health emergency, most autistic children and young people received support (e.g., assessment, intervention sessions) from healthcare professionals online with promising outcomes but evidence lacking to establish accuracy, validity, and long-term implementation (Ellison et al., 2021; Stavropoulos et al., 2022). Virtual reality has also been used to support social, emotional, and behavioural development via realistic scenarios in a safe virtual environment supervised by a healthcare professional with encouraging outcomes for its safety and usability (Bellani et al., 2011; Ghanouni et al., 2019; Malihi et al., 2020). Finally, robots (predominantly humanoid) have mainly been used as mediators of social and communication skills in autistic children and young people in sessions with a healthcare professional with promising albeit questionable outcomes for its clinical effectiveness (Begum et al., 2016; Diehl et al., 2012; Kouroupa et al., 2022; Marino et al., 2020; Wood et al., 2021). Although a recent meta-analysis concluded that humanoid robots were mostly clinically effective in autism clinics with young autistic children aged 4–7 years to support the development of social and communication skills, the meta-analysis was conducted with 12 randomised controlled trials of variable quality (Kouroupa et al., 2022).

Though there are a range of technological approaches, alonsgide the evidence base needed to promote use, there are various child and family factors that are likely play a role in the decision-making processes about their adoption. There is evidence that the child’s age, age of diagnosis, and parents’/carers’ age may direct decisions (Wilson et al., 2018). In addition, the pace of the child’s development, co-occurring conditions, autism symptoms, presence of aggressive challenging behaviour as well as recommendations by others, research evidence, the cost, availability and accessibility of support programmes, parents’/carers’ perception and understanding of autism, the parenting style and their educational level have been reported to influence autism support choices (Carlon et al., 2013; Grant et al., 2016; Dinora et al., 2017; Edwards et al., 2018; Hebert, 2014). However, there is scarce evidence related to the factors that may influence the decision making of families about the use of technology devices as a medium in autism support programmes specifically.

The current study, based on a survey methodology with open and closed responses, aimed to contribute to a better understanding of parents’/carer’s knowledge, interests, and preferences about the use of technology devices in autism. Such knowledge would provide the autistic community with a more comprehensive insight about the added value of technology devices in autism support programmes which could inform the evidence-based support and services for autistic children and young people. Specifically, the study aimed to: (1) identify what technology devices parents/carers were most aware of, their interest towards these and prior level of engagement; (2) understand the most and least preferred technology device parents/carers would choose for their child to engage in an autism support programme; and (3) report the reasoning behind any choice.

## Methods

### Participants

Parents/carers of autistic children and young people were invited to complete the online survey if they had a child (up to and including 18 years old) with or without additional needs (i.e., intellectual disability). Families with children and young people on a waiting list for an autism diagnosis or potential autism were also eligible to complete the study as we deemed it important to glean their preferences too as potential early adopters. In total, 280 parents/carers accessed the survey. After excluding incomplete survey responses (> 70% of missing data), the final sample comprised of 267 participants. Most parents/carers (*n* = 208) were based in the United Kingdom of which 184 were living in England, 11 in Wales, seven in Northern Ireland and six in Scotland. Fifty-nine families were living elsewhere including Europe (*n* = 31) [e.g., Greece (*n* = 19), Cyprus (*n* = 7), Slovakia (*n* = 2), Spain (*n* = 1), Germany (*n* = 1), and Republic of Ireland (*n* = 1], the United States of America (*n* = 19), Australia (*n* = 6), Canada (*n* = 2) and Malaysia (*n* = 1). Autistic children and young people had a mean age of 8.70 years (SD = 4.02, range: 2–18 years) and 73% were male (*n* = 173). The mean age of autism diagnosis was 5.5 years (SD = 3.23; range: 1–17 years). A number of additional support needs were reported (in hierarchical order) including intellectual disability, anxiety, sleep problems, attention deficit hyperactivity disorder, communication problems (e.g., selective mutism, dyslexia, speech delay), chromosomal disorders, eating disorders, epilepsy, dyspraxia, and hearing problems (Table 1). Mostly mothers responded to the survey (*n* = 241, 91%). The mean age of parents/carers was 38.88 years old (SD = 7.98, range: 22–68). About 46% (*n* = 124) of parents/carers had other educational qualifications including GCSEs, A/AS levels, or foundation degree and 31% were working part-time (*n* = 81). Table 1 shows participant demographic information.

**Table 1:** Demographic characteristics of children and parents/carers (*n* = 267)

Characteristic	*N* / Mean (range)	% / SD
Child lives in the UK	208	78
Child lives outside the UK (i.e., Europe, US, Australia, Canada)	59	22
Child is White	238	89
Child is Mixed/Multiple ethnic groups	11	4
Child is Black/African/Caribbean/Black British	5	2
Child is Asian/Asian British	7	3
Child is from other ethnic group	6	2
Child is male	193	73
Child is female	69	26
Child is non-binary	3	1
Child prefers not to say	2	< 1
Child has an autism diagnosis	233	88
Child awaiting diagnosis	22	8
Child has suspected autism	12	4
Child has intellectual disability	100	38
Number of additional needs (mean, range, SD)	1.2 (0–6)	1.5
Child is speaking fluently	186	70
Child is learning to speak	60	22
Child is non-verbal	21	8
Respondent is mother	241	91
Respondent is father	12	4
Respondent is carer (e.g., sibling, foster)	14	7
Respondent has other qualifications (e.g., GCSEs, A/AS levels, foundation degree)	124	46
Respondent has Undergraduate degree	63	24
Respondent has Postgraduate degree	52	19
Respondent has Doctorate/PhD	8	3
Respondent prefers not to say	20	8
Respondent works part-time	81	31
Respondent works full-time	56	21
Respondent is a full-time carer	54	20
Respondent is unemployed not looking for work	38	14
Respondent has other commitments (e.g., retired, student, career break)	25	9
Respondent is disabled	3	1
Respondent prefers not to say	9	4
Family income up to £40,000	168	63
Family income above £40,000	99	37

### Design

This was a survey with open and closed responses (e.g., “Where do you live?”) and open-ended questions (e.g., “Why would you most like your child to have a session with a robot?”). Consistent with the Checklist for Reporting Results of Internet E-Surveys (CHERRIES) guidelines (Eysenbach, 2004, 2012) (Supplementary material), this online survey was designed with input from three parents of autistic children identified via an online parenting group.

### Materials

The online survey included 54 items over 24 pages (1 to 4 questions per page). The survey started with a definition about use of technology devices in an autism support programme to introduce the topic to respondents. It collected detailed demographic and clinical information about the family and the child including age, ethnicity, gender, family income, autism diagnosis of the child. Parents/carers responded to a series of questions about pre-specified technology devices they were aware of, interested to use, previous engagement, the most/least preferred technology device, and the reasoning behind their most and least preferred choice. Following review of the literature and input in the study design from three parents of autistic children acting as experts by experience, the following technology devices were included: smartphone, iPods, tablets, virtual reality, robot. Other and none were also included as options to participants. Sample questions are: “Which technology-based intervention have you heard about for autistic children?” (Multiple responses) and “Which alternative would you LEAST like your child to take part in?” (Single response). Parent’s/Carer’s responses are presented by age group.

### Procedure

The study received ethics approval from the Health, Science, Engineering and Technology Ethics Committee at the University of Hertfordshire (Ref number: LMS/PGR/UH/04164). The participant information sheet and consent form were embedded in the online survey that was hosted by Qualtrics.com. Answers were automatically entered into the Qualtrics database upon completion of a questionnaire. The survey was live for 14 weeks. It was launched online in May 2020 and relaunched in October 2020 for a month. The survey was advertised via autism-specific charities and organisations and social media (i.e., Facebook, Instagram). A number of UK and international autism specific third sector organisations and parenting groups were approached to promote the survey. Due to the ongoing COVID-19 pandemic, most declined promotion of the study link at this challenging time because of a mix of organisational factors or competing research. Two organisations outside of the UK and one in the UK accepted to promote the survey online once without further reminders.

All participants entered the online survey voluntarily, and their participation was pseudonymised; participants created a personal identifier (PID) in case they wanted to delete their responses from the survey later. The survey was completed in one sitting within approximately 20 min for all participants. A review of the IP addresses of those who responded showed that no participants responded to the questionnaire repeatedly. Participants were able to review and change their responses via clicking the back button. There was no financial incentive to complete the survey. All Internet Protocol (IP) addresses and PIDs of participants were deleted when data were extracted for analysis.

### Statistical Analysis

Descriptive statistics were used to describe the sample characteristics, their knowledge, interest, and preferences about technology devices in autism support programmes among three age groups (e.g., 2–5 years old, 6–12 years old, 13–18 years old) because evidence shows that these age groups use technology differently (Hedges et al., 2018; Kuo et al., 2014). The correlation between parent/carer (i.e., age, ethnicity, education, employment status and financial status) and child characteristics (i.e., gender, age, age of diagnosis, country of living, additional diagnoses, speaking skills) with technology devices was explored. Statistical analysis was performed using IBM Statistical Package for Social Sciences (IBM SPSS. version 26.0). Parents’/carers’ open-ended questions were analysed using content analysis in NVivo12. An inductive approach was followed to develop the coding scheme by the first author. The first author initially organised the data in broad categories (i.e., most vs least preferred) and kept adjusting the coding scheme during the line-by-line coding process. After that, the coding scheme was refined creating sub-categories of data. During the second round of coding, 20% of the data were coded by an MSc student (kappa = 0.78) to review alignment of coding to overall thematic description. The final coding scheme was reviewed by the research team. Quantitative data from all participants are presented for the whole sample of participants because there was no divergence of views per age group.

## Results

### Knowledge, Engagement, and Interest in Technology Devices

Parents/carers were asked to report which technology device they know that can be used with autistic children and young people in support programmes (Table 2). For all age groups, parents/carers reported they were mostly aware of tablets followed by none of the listed technology devices.

**Table 2:** Number of parents/carers aware of technology devices in autism support programmes

Technology device	Young children 2–5 years old *n* = 71	Children 6–12 years old *n* = 149	Young people 13–18 years old *n* = 47
	*n* (%)	*n* (%)	*n* (%)
Smartphone	28 (39)	50 (34)	16 (34)
iPod	9 (13)	17 (11)	5 (11)
Tablet	53 (75)	86 (58)	25 (53)
Virtual reality	5 (7)	20 (13)	8 (17)
Robot	12 (17)	20 (13)	4 (9)
None	15 (21)	56 (38)	19 (40)

Parents/carers were asked to report which technology devices their children have engaged with as part of an autism support programme (Table 3). For all age groups, autistic children and young people had been engaged with none of the listed technology devices. About one third of parents/carers reported autistic children and young people had engaged with a tablet. This was a multiple-choice option including smartphone, iPod, tablet, virtual reality headset, robot, none, other. No parents/carers selected “Robots” or “Other”.

**Table 3:** Number of parents/carers whose children engaged with technology devices in autism support programmes

Technology device	Young children 2–5 years old *n* = 71	Children 6–12 years old *n* = 149	Young people 13–18 years old *n* = 47
	*n* (%)	*n* (%)	*n* (%)
Smartphone	14 (20)	28 (19)	11 (23)
iPod	4 (6)	2 (1)	1 (2)
Tablet	28 (39)	26 (38)	16 (34)
Virtual Reality	-	3 (2)	1 (2)
None	35 (49)	88 (59)	29 (62)

Finally, parents/carers were asked to report which technology device they would be most interested in using with their autistic children as part of a support programme (Table 4). For all age groups, parents/carers reported they were mostly interested in tablets. Parents/carers of young autistic people aged 13–18 years reported an interest in the use of smartphones in an autism support programme.

**Table 4:** Number of parents/carers interested in technology devices in autism support programmes

Technology device	Young children 2–5 years old *n* = 71	Children 6–12 years old *n* = 149	Young people 13–18 years old *n* = 47
	*n* (%)	*n* (%)	*n* (%)
Smartphone	26 (37)	70 (47)	31 (66)
iPod	-	-	-
Tablet	65 (92)	124 (83)	40 (85)
Virtual Reality	10 (14)	40 (27)	16 (34)
Robot	14 (20)	28 (19)	15 (32)
None	3 (4)	10 (7)	1 (2)

### Preferences About Technology Devices

Among the list of seven different technology devices, parents/carers were asked to report their most and least preferred choice they would use with their autistic child. For all age groups, the most preferred technology device was the tablet (Table 5).

**Table 5:** Number of parents/carers who mostly preferred technology devices in autism support programmes

Technology device	Young children 2–5 years old *n* = 71	Children 6–12 years old *n* = 149	Young people 13–18 years old *n* = 47
	*n* (%)	*n* (%)	*n* (%)
Smartphone	12 (17)	24 (16)	11 (23)
iPod	1 (< 1)	0	0
Tablet	48 (68)	86 (58)	24 (51)
Virtual Reality	3 (4)	15 (10)	6 (13)
Robot	1 (< 1)	10 (7)	4 (9)
None	4 (6)	10 (7)	0
Other (computer/laptop)	2 (3)	4 (3)	2 (4)

Table 6 presents the number of parents/carers who reported their least preferred technology device to use with their autistic child as part of an autism programme. For all age groups, the least preferred technology device was a virtual reality headset followed by robots.

**Table 6:** Number of parents/carers who least preferred technology devices in autism support programmes

Technology device	Young children 2–5 years old *n* = 71	Children 6–12 years old *n* = 149	Young people 13–18 years old *n* = 47
	*n* (%)	*n* (%)	*n* (%)
Smartphone	7 (10)	14 (9)	2 (4)
iPod	4 (6)	19 (13)	8 (17)
Tablet	7 (10)	8 (5)	4 (9)
Virtual Reality	29 (41)	37 (25)	8 (17)
Robot	14 (20)	45 (30)	10 (21)
None	8 (11)	24 (16)	13 (28)
Other (computer/laptop)	2 (3)	2 (1)	2 (4)

### Reasons for Most and Least Preferred Technology Device

Tablets were mostly preferred because autistic children and young people found them easy to control, are easily available in most households and subsequently is a familiar and portable device. On the contrary, respondents reported that a virtual reality headset was the least preferred technology device due to the sensory overload that autistic children and young people might experience in a virtual reality session. The immersive environment of virtual reality appeared to be of concern to parents/carers who expressed that it is an unnatural environment that will likely confuse and upset autistic children and young people. On the other hand, a small number of parents/carers thought the immersive environment of virtual reality might be beneficial because autistic children and young people might be able to remain focused longer. Robots were considered to be less popular compared to other technology devices due to their size, lack of space, immobility and cost. Respondents also described robots as being cold, and impersonal. Robots were an unknown technology device for most parents/carers to consider using with their autistic children. Finally, iPods were repeatedly described as outdated and impractical technology device. See Table 7 with sample quotes per technology device and the reasons for parent’s/carer’s preferences.

**Table 7:** Reasons provided for most and least preferred technology devices (for all age groups) in autism support programmes

Technology device	Reason for most preferred	Quotes
Tablet	Ease of control (n* = 49)	“He is able to control the iPad with his finger which results in less frequent melt downs as he struggles with tv because he cannot tell us what he wants on.”, “Easier to use”, “Uses her iPad very well”
	Availability (*n* = 49)	“It is the most accessible option. He already has a tablet and enjoyed doing his home schoolwork on it during lockdown.”, “He has a tablet”
	Familiarity (*n* = 45)	“She is comfortable using one and finds it easier to communicate this way…we could easily integrate it.”, “He is used to that technology.”, “It is a familiar piece of equipment.”
	Portable (*n* = 15)	“Convenient to carry around”, “Easy to carry anywhere”
Smartphone	Accessibility (*n* = 29)	“We both own one and he uses this all the time”, “She has one with her all the time”
	Convenience (*n* = 19)	“Because it’s easy to use and has a great variety of interesting activities to be engaged in, with the aim of education or just enjoyment and fun.”, “Easier to use”
Virtual reality	Immersive technology (*n* = 17)	“I think that he would benefit from feeling completely immersed in the experience as can be distracted easily.”, “Shuts off from the world and enters another”
	Previous experience (*n* = 7)	“My son has had only one experience of using a virtual headset but really seemed to enjoy it.”, “He would engage with it as he has asked previously for a VR headset.”
Robot	Engaging nature (*n* = 10)	“Think would be a good middle ground between human and learning but a ‘friend’ too.”, “He would like the movement of the robot”
	Child’s interest in technology (*n* = 7)	“He enjoys robots and robotics and would likely engage with this well.”, “She loves technology and loves the idea of robots”
None	Technology addiction (*n* = 4)	“He can get fixated with technology and then it’s hard to get him to do anything else after.”, “We currently limit the use of screen time.”, “addicted to technology”
	Personal view about technology (*n* = 4)	“I don’t always agree that technology is the best for my child.”, “I prefer human intervention”, “Prefer no devices”
iPod	Used in PECS (*n* = 1)	“Used one for PECS (Picture Exchange Communication System).”
**Technology-based intervention**	**Reason for least preferred**	**Quotes**
Tablet	Age of the child (*n* = 15)	“He becomes addicted to screens.”, “I don’t want him to use technology for learning at this age”
Smartphone	Age of the child (*n* = 16)	“Phones are not always meant for kids.”, “Smart phones open your child up to bullying and at 9 I feel my child is too young for one.”, “Smartphones already create addiction to him”
	Lack of control (*n* = 3)	“Not as easy to monitor as an iPad”, “I cannot control the use of a smartphone if he has one.”
	Screen size (*n* = 4)	“Screens are too small.”
Virtual reality	Immersive technology (*n* = 73)	“I would be concerned about the effects on him emotionally blurring lines between reality and virtual reality…he may find it difficult to separate it using this kind of technology.”, “Unrealistic”, “Sensory overload”, “too complicated / overwhelming”
	Cost (*n* = 3)	“They are expensive to buy.”
Robot	Physical appearance (*n* = 32)	“Scary”, “Unnatural”, “Too intimidating”, “Odd”
	Unknown technology (*n* = 21)	“It is new technology. I have no information on how this would work.”, “Unfamiliar”, “New”, “Unknown territory”
	Cost (*n* = 5)	“I assume it would be expensive.”, “Cost”
	Structural characteristics (*n* = 3)	“Inconvenient to carry around.”, “Space”, “Size”
iPod	Old-fashioned (*n* = 32)	“Screen is too small”, “Not practical”, “He would lose concentration because he relies on visual aids.”, “Very outdated”

*n reflects the number of counts of references across participants

## Discussion

This cross-sectional study explored parents’/carers’ knowledge, engagement, interest, and preferences about technology devices with autistic children and young people as part of an autism support programme. Parents/carers of autistic children and young people were aware of, interested in and mostly preferred the use of tablets across all age groups because of their convenience and ease of use. Other technology devices such as virtual reality and/or a robot raised several concerns due to their perceived characteristics and appearance, respectively. The use of virtual reality and/or robots in autism support programmes is a relatively new topic with unclear outcomes due to small scale and proof of concept studies (Dechsling et al., 2021; Kouroupa et al., 2022).

The increased knowledge and preference of families to introduce tablets to autistic children and young people as part of an autism support programme might indicate parents’/carer’s attitudes towards controlled and supervised use of tablets by their child as well as the child’s interest to access tablets as they get older. National data suggest most households have access to tablets, complimented by schemes aimed at supporting those who do not such as disadvantaged households during the coronavirus pandemic to facilitate remote school attendance (Department for Education, 2022; Office for National Statistics, 2018). Although, it is encouraging that tablets have attracted the attention of families of autistic children and young people, the empirical evidence of their effectiveness is limited and debatable in the autism literature (Fletcher-Watson et al., 2016; Maglione et al., 2012). In addition, the growing knowledge and preference towards tablets might indicate that parents’/carer’s attitudes are likely to be shaped by their child’s use of technology at home or in school. However, previous work has reported parents’/carer’s concerns about increased screen time in autistic children as well as that their autistic children were not sharing what they do with the tablet (Laurie et al., 2019). Nonetheless, in this study, the use of a tablet was reported to be the most preferred technology device across all age groups due to convenience. Technologies such as a robot or a virtual reality headset raised concerns in parents/carers of autistic children and young people with their design and appearance and their special characteristics (e.g., claustrophobic).

Within this context, although autistic children and young people show some affinity towards technology, there is limited evidence about their effectiveness and no longitudinal outcomes in relation to social and communication, educational or other skills (Costescu et al., 2014; Kouroupa et al., 2022; Sandbank et al., 2020; Soares et al., 2021). There is evidence on the effect of new technology devices (e.g., smartphones, virtual reality, robots) showing that more and more autistic children and young people have been exposed to different technology devices in an experimental context (Fletcher-Watson et al., 2016; Grynszpan et al., 2014; Hedges et al., 2018; Pennisi et al., 2016; Sandbank et al., 2020). Interestingly, the use of virtual reality and robots appears to be a promising avenue to support education and the social and communication skills of young autistic children (Kouroupa et al., 2022; Zhang et al., 2022) but is less known by parents/carers suggesting that normalisation and accessibility of these support approaches is yet to be progressed for most benefit. The lack of available and validated autism specific support after diagnosis remains a significant problem and a key drive to accessing more and more new approaches.

Notably, 70% of autistic children and young people in the study were reported to speak fluently while 38% of autistic children and young people were reported to have an intellectual disability. These factors - verbal fluency and variable support needs – likely influence the use of technology devices by parents/carers, suggesting that technology devices could be primarily used for leisure rather than in a structured autism support programme such as the use of tablets for Augmentative and Alternative Communication (AAC). However, the focus of this study was on capturing the overall knowledge and preferences of technology devices use rather than on specific application such as AAC, which may differ based on individual support needs.

Developing a better knowledge and understanding of families’ preferences around technology devices in autism support programmes and the factors associated with decision-making would facilitate healthcare professionals to inform families of the range of evidence-based support available as well as to inform researchers working on evidence of the benefit to be more attuned to parent/carer information needs and preferences. It is important to ensure that families have easy access to guidance and support when they seek information about the most optimal support programme on behalf of their autistic child. Enhancing the provision of evidence-based support programmes for autistic children and young people may be significantly improved by integrating technology devices (i.e., tablets) during a session. Technology devices could be an accessible, interactive, and personalised platform that can be tailored to meet the individual support needs of the autistic population. For example, the use of applications and software designed for skill development, communication, and behavioural tracking would allow a consistent, repetitive and secure environment leading to quality data across multiple environments including clinics, home, and school to monitor progress.

The study findings of the most preferred technology device with autistic children and young people have a number of implications for the design of innovative approaches and future research. New technologies for autistic children and young people need to be accessible to children, young people and their families and competitive against current technologies autistic children and young people use. Tablets could facilitate the remote delivery of autism support programmes, making evidence-based practices more accessible and inclusive to those in underserved areas through telehealth services. However, (healthcare) professionals and families need to collaborate to facilitate the implementation of a holistic and personalised support programme through a tablet ensuring high-quality care is provided to autistic individuals. Additionally, data collected through tablet applications could inform the decision-making and the need of future adjustments to the healthcare plan, leading to targeted and effective support programmes. Utilising technology devices for data-driven decision-making and early screenings can significantly improve outcomes. For example, the use of tablets in autism support programmes, remotely or not, needs to be further explored in relation to their effectiveness during a session either controlled by a trained (healthcare) professional or a parent/carer depending on the context of a session (e.g., home, clinic, school). The level of awareness on the way these technology devices could be used and previous experience of using and/or how these devices would be used as an educational, behavioural or other support medium should also be explored in more depth in the future. Future research should focus on developing an evidence-based on the way technology devices are being used as part of an autism support programme and monitor their availability to autistic children and young people as technology evolves and change over time. It is equally important to systematically examine the preferences of autistic children and young people for different types of technology devices, the focus and format of autism support programmes and the specific support needs of autistic people as there is some evidence that access to autism specific support is decreasing as children get older (Gibson, Kaplan, & Vardell, 2017). Finally, advocating for supportive policies and promoting cultural competence in autism support programmes ensures that care is inclusive and equitable for all individuals. By combining traditional evidence-based approaches with innovative technology devices such as a tablet, the quality and accessibility of care for autistic individuals can be greatly enhanced.

### Limitations

The study findings should be interpreted in light of its limitations. Among the limitations of this work is that autism diagnosis was parent/carer-reported and not verified with a healthcare professional. In addition, there were 8% of children and young people on a waiting list for an autism diagnosis and 4% of children and young people with suspected autism based on parent/carer report. This may have introduced some nuance in the precise description of the study sample. It is also the case that results rely on parent/carer reports of technology preferences rather than the child’s attitudes to technology devices. In the context of the study, this was however important as the aim was to look at gatekeeper preferences and knowledge. Respondents were mostly of a White heritage and mothers so were not representative of the UK profile. This broadly reflects the time at which the study was conducted (i.e. pandemic context) alongside wider under-representation of fathers in autism research. As the study was online, the respondents clearly use and have access to technology too. It is plausible that they may have a positive view about technology devices to some degree. Future work will therefore benefit from understanding enablers of technological devices in those with access and or engagement barriers. There is also a temporal gap between the time the study was conducted and its publication which suggests careful interpretation of data. Considering post pandemic attitudes would be useful to understand how a crisis may have driven digital health and intervention attitudes. It would also be useful to understand the extent to which the findings reflect parental trends in the main or autism specific by way of including a comparator group of neurotypical youth. In addition, the value of this work is limited by the lack of a comparison group that could have enhanced the interpretation of the data about the knowledge and preferences of parents/carers of autistic children and young people about technology devices. It is likely that the increased use of smartphones in autistic children aged 6–12 years (50%) might reflect the widespread introduction of technology devices in our lives and not their value to be handled as an attractive technology device to engage in an autism support programme. For example, recent data indicated that 90% of children own a mobile phone from the age of 11 (House of Commons (2024). Finally, despite the definition of technology-based interventions provided at the start of the survey, it is likely that parents’/carer’s preferences may have been influenced by their own understanding of technology use at home, especially in the absence of specific examples related to technology device use in an autism support programme in the free-text area (Smith & Boyles, 2012). Autistic children and young people often use tablets for relaxation or fun but not as part of a support programme that might require the control of the device by an adult/trained healthcare professional (Achtypi et al., 2023).

## Conclusion

The findings of this study highlight that families selected tablets as the most preferred technology device to use with autistic children and young people during a support programme. Based on parents’/carers’ responses in the survey, we suggest that technology devices have attracted the attention of families who might use technology devices not only for leisure but also as part of supporting learning of new skills. Future research will benefit within the context of prioritising digital and technological innovation in health to consider how to optimise use and evidence on impact in autism support programmes. Inclusion of autistic children and young people themselves in prioritisation of research and the design and implementation of interventions will accelerate knowledge on what work and for whom, and in what circumstances,

## Electronic supplementary material

Below is the link to the electronic supplementary material.

Supplementary Material 1
